# Integrative Single‐Cell and Bulk Transcriptomics Identifies a Proliferative Malignant State and Supports a Functional Contribution of TROAP in Osteosarcoma

**DOI:** 10.1155/ijog/1407947

**Published:** 2026-10-14

**Authors:** Peilei Jiao, Xiuqi Chen, He Wei, Zhihua Sun, Miao Lu, Ye Bai, Wenjin Zhang, Dexin Wang

**Affiliations:** ^1^ Department of Orthopedics The 962nd Hospital of the Chinese People′s Liberation Army Joint Logistics Support Force Harbin Heilongjiang China; ^2^ Department of Clinical Medicine Heilongjiang Nursing College Harbin Heilongjiang China; ^3^ Graduate School Mudanjiang Medical University Mudanjiang Heilongjiang China mdjmu.cn; ^4^ Department of Orthopedics The Second Affiliated Hospital of Heilongjiang University of Chinese Medicine Harbin Heilongjiang China hljucm.edu.cn

**Keywords:** copy-number variation, LASSO, malignant cell state, metastasis, osteosarcoma, single-cell RNA sequencing, TROAP, tumor microenvironment

## Abstract

**Background:**

Osteosarcoma contains heterogeneous malignant‐cell states whose clinical relevance is difficult to infer from small single‐cell cohorts.

**Methods:**

We reanalyzed six treatment‐naive osteosarcomas from GSE162454, identified CNV‐supported malignant osteoblastic cells, and characterized a recurrent proliferative/mitotic state using patient‐paired pseudobulk analysis and program‐selection sensitivity tests. A 50‐gene module was evaluated for association with metastatic occurrence in 53 pretreatment tumors from GSE21257. Candidate ranking was audited across transparent weighting scenarios, and TROAP was depleted with two independent siRNAs in HOS and U2OS cells.

**Results:**

Of 52,489 input barcodes, 39,063 singlets were retained, including 10,456 CNV‐supported malignant osteoblastic cells. M6 comprised 725 cells and was detected in all six tumors. It ranked first in six of eight program‐selection scenarios but fell to fifth when G2M checkpoint and E2F targets were jointly excluded, supporting its interpretation as a proliferative/mitotic state. Its weighted module was higher in metastatic tumors (*p* = 0.0147; apparent AUC = 0.704), but the association was attenuated after macrophage adjustment (OR = 1.646, 95% CI 0.649–4.170; *p* = 0.2937), whereas the macrophage score remained inversely associated with metastasis (OR = 0.251, 95% CI 0.081–0.773; *p* = 0.0161). TROAP was strongly enriched in M6 and supported by paired pseudobulk analysis, but it was not the top‐ranked candidate under every weighting scenario. TROAP depletion reduced colony formation, overall wound closure, and Transwell migration and invasion in both cell lines.

**Conclusion:**

A recurrent proliferative/mitotic malignant state is detectable across the six osteosarcomas and yields a bulk expression program associated with metastatic occurrence, although this association is sensitive to macrophage composition. TROAP is supported as a functional contributor to osteosarcoma cell phenotypes rather than an established driver of the state or an independently validated clinical marker.

## 1. Introduction

Osteosarcoma is the most common primary malignant bone tumor in children and adolescents. Multiagent chemotherapy combined with complete surgical resection has transformed outcomes for localized disease, yet metastatic or recurrent osteosarcoma continues to carry a poor prognosis and has not experienced a comparable therapeutic advance [1, 2]. The biological obstacle is not simply a shortage of candidate genes. Osteosarcoma genomes are characterized by structural complexity, widespread copy‐number alteration, chromothripsis, and pronounced intertumoral and intratumoral heterogeneity, making a single recurrent initiating lesion difficult to nominate [3–5]. A clinically useful target may therefore be encoded by a recurrent malignant‐cell state rather than by a ubiquitous DNA alteration.

Single‐cell and spatial transcriptomic studies have begun to resolve this problem. Human osteosarcomas contain malignant osteoblastic cells interwoven with myeloid, lymphoid, fibroblast, endothelial, perivascular, and osteoclast‐lineage populations [6–8]. These compartments are not static. Treatment, anatomical site, and disease progression reshape both malignant programs and immune‐stromal niches [9–11]. Recent analyses further implicate exhausted T‐cell states, RNA‐regulatory programs, and context‐dependent cell–cell signaling in osteosarcoma heterogeneity [12–14]. Cross‐dataset communication analyses also connect tumor–microenvironment signaling with metastatic behavior [15]. Nevertheless, two translational gaps remain. First, malignant subpopulations identified in a small single‐cell cohort may represent patient‐specific expansions rather than reproducible states. Second, a state‐defining marker can be statistically prominent without being associated with patient outcome or having a functional contribution to malignant behavior.

Bulk clinical cohorts can help bridge the first gap by testing whether a single‐cell‐derived program is detectable across independent pretreatment tumors. GSE21257 is particularly relevant because it links gene expression in 53 pretreatment biopsies to development of metastasis within 5 years [16]. Recent integrative osteosarcoma studies illustrate the value of combining bulk and single‐cell evidence but also the frequency with which small cohorts are used to derive multigene signatures [17, 18]. Its modest size therefore makes unconstrained genome‐wide model building vulnerable to overfitting. Candidate derivation must be separated from clinical testing; patient‐level replication should be incorporated within the single‐cell discovery cohort, and predictive performance should be estimated with all selection steps contained inside resampling.

Here, we used GSE162454 to construct a quality‐controlled osteosarcoma atlas, infer CNV‐supported malignant osteoblastic cells, and identify a recurrent proliferative/mitotic malignant state. We then required concordant cell‐level and patient‐pseudobulk evidence before evaluating the resulting program in GSE21257. Transparent sensitivity analyses integrated malignant‐state specificity, bulk association, discrimination, and selection stability. TROAP was selected as one of several high‐priority candidates for functional testing rather than as the unambiguously top‐ranked gene. TROAP encodes trophinin‐associated protein, a mitosis‐associated protein linked to proliferation and motility in several cancers, including soft‐tissue sarcoma, but its functional relevance in osteosarcoma has remained insufficiently defined [19–21]. We therefore tested whether TROAP depletion alters clonogenic growth and wound closure, migration, and invasion in two osteosarcoma cell lines. This design moves from cellular‐state discovery to cross‐cohort association and focused biological testing without treating exploratory prediction as clinical proof.

## 2. Materials and Methods

### 2.1. Study Design and Datasets

The study integrated single‐cell discovery, cross‐cohort bulk‐tumor evaluation, and in vitro functional testing. Single‐cell RNA‐sequencing data were obtained from GSE162454, comprising six treatment‐naive primary human osteosarcoma specimens profiled with the 10x genomics platform [6]. GSE21257 contained 53 pretreatment osteosarcoma biopsies, including 34 patients who developed metastasis within 5 years and 19 who remained metastasis‐free for at least 5 years [16]. The single‐cell cohort was used to define the tumor ecosystem, identify CNV‐supported malignant osteoblastic cells, resolve malignant heterogeneity, and derive candidate markers. The bulk cohort was used to assess whether the single‐cell‐derived program was associated with metastatic occurrence and to compare candidate genes; it was not treated as cell‐type‐specific validation.

### 2.2. Single‐Cell Data Import and Quality Control

The six filtered feature‐barcode matrices supplied by GEO were imported into R and converted to Seurat objects. Genes detected in fewer than three cells were excluded. For each barcode, the number of detected genes, total unique molecular identifiers, and the fractions of mitochondrial, ribosomal, and hemoglobin transcripts were calculated. Because filtered rather than raw droplet matrices were available, ambient‐RNA estimation was not attempted. Quality‐control limits were estimated separately for each patient using median absolute deviation‐based thresholds. The minimum detected‐gene cutoff was constrained to at least 200 genes; upper gene‐count and UMI limits used five upper median absolute deviations; and the mitochondrial limit used three upper median absolute deviations constrained between 10% and 25%. Candidate singlets were then evaluated using scDblFinder in a sample‐aware manner, and only final singlets were retained [22].

### 2.3. Normalization, Integration, Dimensionality Reduction, and Clustering

RNA counts were log‐normalized for expression visualization and differential testing. Variance stabilization was performed with SCTransform v2 using glmGamPoi when available, mitochondrial percentage as a regression covariate, and 3000 variable genes. Principal‐component analysis generated 50 components. Patient‐associated technical variation was reduced with Harmony using patient identity as the integration variable [23]. The first 30 Harmony dimensions were used to construct the neighbor graph and uniform manifold approximation and projection (UMAP) embedding. Graph‐based clustering used a resolution of 0.6. Harmony coordinates were used only for embedding and clustering; the RNA assay was retained for marker testing. The workflow followed current scalable single‐cell integration principles while preserving uncorrected expression for biological comparisons [24].

### 2.4. Major‐Cell‐Type and Detailed‐State Annotation

Major lineages were annotated through concordance among canonical marker expression, UCell signature scores, cluster‐level markers, and SingleR predictions when the reference resource was available [25]. Marker programs covered osteoblastic/mesenchymal, myeloid, T/NK, B/plasma, fibroblast, endothelial, pericyte, and mast‐cell identities. Cluster assignments were reviewed in an editable annotation table before acceptance. Detailed states were subsequently assigned using lineage‐restricted markers and cluster‐specific transcriptional evidence. Because single‐cell labels can compress continuous or mixed phenotypes, detailed annotations were treated as descriptive transcriptional states.

### 2.5. Transcriptome‐Derived Copy‐Number Inference and Malignancy Assignment

Malignant osteoblastic cells were distinguished from nonmalignant stromal cells using inferCNV. T/NK, B/plasma, myeloid, and endothelial populations (4854 cells in total) served as references. Osteoblastic/mesenchymal cells were the principal candidate population, whereas fibroblasts and pericytes were retained as observation groups because their mesenchymal identity could make them unsuitable normal references and could otherwise attenuate tumor‐associated expression‐derived CNV signals. InferCNV used a cutoff of 0.1 with denoising enabled and hidden Markov model calling disabled. A per‐cell CNV score was calculated as the mean squared deviation of the smoothed expression profile from the reference baseline. The reference‐cell 95th percentile (0.02019) was used as the cell‐level support threshold. A cell was classified as CNV‐supported malignant only when it had both osteoblastic/mesenchymal lineage identity and a CNV score above this threshold. Elevated CNV scores in fibroblasts or pericytes were audited but did not trigger reassignment because RNA‐derived CNV elevation alone was not considered proof of malignancy. Chromosome‐level summaries were calculated by averaging gene‐level deviations within each chromosome and malignant subcluster. These measurements represent broad expression‐derived patterns rather than direct DNA copy‐number calls [3, 26].

### 2.6. Malignant‐Cell Reclustering and Definition of the M6 Proliferative/Mitotic State

CNV‐supported malignant osteoblastic cells were reprocessed independently so that immune and stromal compartments did not dominate their latent structure. SCTransform, principal‐component analysis, Harmony integration, UMAP, graph construction, and clustering were repeated. Forty principal components were calculated, the first 25 Harmony dimensions were used, and clustering was performed at a resolution of 0.5. Six Hallmark programs‐E2F targets, G2M checkpoint, epithelial‐mesenchymal transition, hypoxia, glycolysis, and MYC targets V1‐were scored using UCell. For each program, mean scores were standardized across malignant subclusters; the composite score was the arithmetic mean of the six standardized scores. Only subclusters containing at least 50 cells and represented in at least three patients were eligible. The highest‐scoring eligible cluster was provisionally designated the M6 proliferative/mitotic state. To assess dependence on program choice, the composite score was recalculated after removing each program separately and after jointly removing G2M checkpoint and E2F targets. This terminology describes the observed transcriptional phenotype and is not a prospectively established clinical risk category.

### 2.7. Differential Expression and Candidate‐Gene Derivation

Markers of the M6 proliferative/mitotic state were evaluated at both cell and patient levels. Cell‐level Wilcoxon tests compared M6 with all other malignant cells. Positive markers required adjusted *p* < 0.05, average log2 fold − change > 0.25, detection in at least 25% of M6 cells, and a detection − fraction difference > 0.10. Patient‐aware pseudobulk profiles were generated by aggregating raw counts separately for M6 and other malignant cells within each patient. The edgeR design was ~patient + cell_state, thereby treating the two cell states from each of the six patients as paired observations. TMM normalization, robust dispersion estimation, and quasi‐likelihood testing were used. Candidates required absolute log2 fold − change > 0.50 and FDR < 0.05 in pseudobulk analysis. Genes supported by both cell‐level and pseudobulk analyses formed the highest‐confidence tier; mitochondrial, ribosomal, hemoglobin, and predefined technical genes were excluded.

### 2.8. Functional Enrichment and Exploratory Trajectory Analysis

Gene Ontology over‐representation analysis was performed with clusterProfiler using high‐confidence markers, and redundant terms were condensed for display. Hallmark gene‐set enrichment analysis used the ranked pseudobulk differential‐expression statistic; normalized enrichment scores and Benjamini–Hochberg‐adjusted *p* values were reported. Slingshot was used only for exploratory transcriptional ordering [27]. M2, the cluster with the lowest original six‐program composite score, was supplied as the starting cluster; no terminal cluster was imposed. Six lineages were inferred, after which the lineage containing M6 (M2‐M8‐M6) was selected post hoc for visualization. Candidate‐gene expression was summarized in 20 equal‐frequency pseudotime bins and row‐standardized. Because the data are cross‐sectional and the displayed lineage was selected for containing M6, pseudotime was not interpreted as independent evidence of temporal progression toward M6.

### 2.9. Processing and Clinical Labeling of GSE21257

The GSE21257 series matrix and GPL10295 platform annotation were imported in R. The supplied matrix was checked for log‐scale comparability. Platform probes were mapped to official gene symbols, yielding 30,549 mapped probes and 24,996 genes after gene‐level consolidation. Clinical labels were fixed before association testing. The final comparison comprised 34 patients who developed metastasis within 5 years and 19 nonmetastatic patients. Audit of the accompanying survival annotations confirmed that all 19 nonmetastatic patients had at least 60 months of recorded follow‐up. Fifty genes selected from the single‐cell analysis were represented on the platform. Principal‐component analysis of the bulk‐expression profiles is shown in Figure S6A.

### 2.10. Cross‐Cohort Evaluation of the M6‐Derived Program

For each GSE21257 sample, expression of each of the 50 mapped genes was standardized across the cohort. All genes were assigned the positive direction. The module score for sample j was S_j = sum_g(w_g z_gj), where z_gj is the standardized expression of gene g and w_g is its normalized single‐cell evidence weight; the 50 weights sum to 1. The complete gene list, directions, weights, and sample‐level scores are provided in Tables S3 and S4. Scores were compared using a two‐sided Wilcoxon rank‐sum test. Discrimination was summarized by the apparent area under the receiver operating characteristic curve with a 95% confidence interval. Individual candidates were examined using limma differential expression, standardized single‐gene logistic regression, and descriptive single‐gene ROC analysis. Genome‐wide *p* values were adjusted by the Benjamini–Hochberg method. Because bulk expression cannot establish that the signal arose specifically from M6 cells, this analysis was termed a cross‐cohort bulk association rather than independent validation.

### 2.11. Penalized Modeling and Internal Validation

Multigene metastatic‐outcome modeling used penalized logistic regression. Performance was estimated using repeated nested five‐fold cross‐validation, with feature filtering, feature selection, and penalty tuning restricted to training folds. The procedure was repeated 20 times. Out‐of‐fold predictions were combined to estimate cross‐validated AUC and its 95% CI. Selection stability was quantified across nested fits and in 100 valid bootstrap fits. This design was chosen because resampling outside the complete selection workflow can substantially overstate performance in small transcriptomic cohorts.

### 2.12. Multievidence Target Prioritization and Sensitivity Analyses

Target prioritization was treated as a transparent sensitivity analysis rather than a claim that one gene was uniformly best. Seven normalized evidence components were used in this order: single‐cell specificity, bulk effect, bulk statistical evidence, single‐gene AUC, LASSO stability, expression specificity, and macrophage‐adjusted robustness. Four revision scenarios were examined. Their respective weights were: balanced, 0.20/0.15/0.15/0.10/0.15/0.10/0.15; single‐cell priority, 0.35/0.10/0.10/0.08/0.10/0.17/0.10; bulk priority, 0.12/0.25/0.25/0.15/0.08/0.05/0.10; and robustness priority, 0.12/0.10/0.10/0.08/0.25/0.05/0.30. Scenario scores were weighted sums of these components. Genes with directionally concordant evidence formed the eligible shortlist. The four scenarios were used to examine ranking stability during revision and were not represented as prospectively prespecified rules. TROAP was selected for functional follow‐up from the Tier A shortlist on the basis of convergent evidence, biological interpretability, incomplete osteosarcoma‐specific characterization, and experimental feasibility; it was not the highest‐ranked gene under every scenario.

To evaluate immune‐composition sensitivity, a macrophage expression score was calculated in GSE21257 and modeled jointly with the M6‐derived module. Both scores were standardized, and odds ratios were reported per one‐standard‐deviation increase. The unadjusted module model and the joint module‐plus‐macrophage model were reported together. Overall survival was explored after median dichotomization of the module score; this analysis was hypothesis‐generating and was not used for target selection (Figure S8C).

### 2.13. Cell Culture and siRNA Transfection

HOS and U2OS human osteosarcoma cell lines were obtained from the American Type Culture Collection (ATCC; Manassas, Virginia, United States; HOS, CRL‐1543; U2OS, HTB‐96). Cell‐line identity was authenticated by short tandem repeat profiling within 6 months before the experiments. Cultures were tested monthly for mycoplasma contamination using the MycoAlert Mycoplasma Detection Kit (Lonza, Basel, Switzerland) and remained negative. Cells were maintained in Dulbecco′s modified Eagle medium (DMEM; Gibco, Thermo Fisher Scientific, Waltham, Massachusetts, United States; cat. no. 11965092) supplemented with 10% (v/v) fetal bovine serum (Gibco; cat. no. 10099141) and 1% penicillin–streptomycin (final concentrations, 100 U/mL penicillin and 100 *μ*g/mL streptomycin; Gibco) at 37°C in a humidified atmosphere containing 5% CO₂.

Small interfering RNAs (siRNAs) were synthesized by GenePharma (Shanghai, China). The duplex sequences were: siTROAP‐1 sense, 5 ^′^‐GCAGAAACCACCGCUCAAU‐dTdT‐3 ^′^, and antisense, 5 ^′^‐AUUGAGCGGUGGUUUCUGC‐dTdT‐3 ^′^; siTROAP‐2 sense, 5 ^′^‐CCAACCCUGUGGCUACAUU‐dTdT‐3 ^′^, and antisense, 5 ^′^‐AAUGUAGCCACAGGGUUGG‐dTdT‐3 ^′^. Cells were seeded in six‐well plates at 3 × 105 cells per well. At 60%–70% confluence, cells were transfected with 50 nM nontargeting control siRNA (siNC), siTROAP‐1, or siTROAP‐2 using Lipofectamine 3000 (Invitrogen, Thermo Fisher Scientific) according to the manufacturer′s instructions. Functional assays were initiated 48–72 h after transfection.

### 2.14. Immunoblotting

Cells were lysed on ice in RIPA buffer (Beyotime Biotechnology, Shanghai, China) supplemented with 1% protease and phosphatase inhibitor cocktail (Roche, Basel, Switzerland). Protein concentrations were measured using a bicinchoninic acid protein assay kit (Thermo Fisher Scientific). Equal amounts of protein (30 *μ*g per lane) were separated by 10% SDS–PAGE and transferred to 0.45‐*μ*m polyvinylidene difluoride membranes (Millipore, Billerica, Massachusetts, United States). Membranes were blocked for 1 h at room temperature with 5% nonfat milk in Tris‐buffered saline containing 0.1% Tween‐20 and incubated overnight at 4°C with anti‐TROAP (1:1000; cat. no. 13019‐1‐AP; Proteintech, Wuhan, China) or anti‐*β*‐actin (1:5000; cat. no. 66009‐1‐Ig; Proteintech). After washing, membranes were incubated for 1 h at room temperature with the appropriate horseradish‐peroxidase‐conjugated secondary antibody (1:5000; Cell Signaling Technology, Danvers, Massachusetts, United States). Signals were developed using enhanced chemiluminescence reagent (Bio‐Rad, Hercules, California, United States), captured with a ChemiDoc imaging system (Bio‐Rad), and quantified using ImageJ (National Institutes of Health, Bethesda, Maryland, United States). *β*‐actin served as the loading control.

### 2.15. Colony‐Formation, Scratch‐Wound, and Transwell assays

For colony‐formation assays, transfected cells were seeded in six‐well plates at 1000 cells per well and cultured for 14 days, with medium replaced every 3 days. Colonies were fixed with 4% paraformaldehyde for 15 min and stained with 0.1% crystal violet for 20 min at room temperature. Colonies containing more than 50 cells were manually counted using a stereomicroscope.

For scratch‐wound assays, cells were grown to approximately 90% confluence in 6‐well plates. A linear wound was generated with a sterile 200‐uL pipette tip, and detached cells were removed by washing twice with phosphate‐buffered saline. Cells were then maintained in serum‐free DMEM. Images were acquired at 0 and 24 h with an inverted phase‐contrast microscope (Olympus, Tokyo, Japan) in three randomly selected fields per well. Wound areas were measured in ImageJ, and wound closure was calculated as [(wound area at 0 h − wound area at 24 h)/wound area at 0 h] × 100%. No proliferation‐matched normalization or pharmacological proliferation blockade was applied; accordingly, this assay was interpreted as overall wound closure rather than a direct measure of migration alone.

Transwell migration and invasion assays were performed in 24‐well chambers fitted with polycarbonate membrane inserts with 8.0‐*μ*m pores (Corning Costar, Cambridge, Massachusetts, United States). For invasion assays, the upper surface was coated with Matrigel (BD Biosciences, San Jose, California, United States) diluted 1:8 in basal medium; migration assays used uncoated inserts. Cells (5 × 104) in 200 *μ*L serum‐free medium were placed in the upper chamber, and 600 *μ*L complete medium containing 10% fetal bovine serum was added to the lower chamber as the chemoattractant. After incubation for 24 h at 37°C, nonmigrated cells were removed from the upper surface with a cotton swab. Cells on the lower membrane surface were fixed with 4% paraformaldehyde for 15 min, stained with 0.1% crystal violet for 20 min, and imaged using an inverted microscope. Cells were counted in five randomly selected fields per insert using ImageJ.

### 2.16. Statistical Analysis and Figure Preparation

All statistical tests were two‐sided unless stated otherwise. FDR was controlled using the Benjamini–Hochberg procedure. Continuous effects are reported with 95% CIs where available. Cell‐culture experiments were performed in three independent biological replicates and are presented as mean ± standard deviation. One‐way analysis of variance followed by Dunnett′s multiple‐comparison test was used to compare siTROAP‐1 and siTROAP‐2 separately with the siNC control. Statistical significance in the experimental figure was denoted as  ^∗∗∗^ *p* < 0.001. Analyses were conducted in R using Seurat, Harmony, scDblFinder, UCell, inferCNV, edgeR, limma, clusterProfiler, fgsea, Slingshot, glmnet, and related Bioconductor packages. Figures were exported as vector PDF and high‐resolution RGB raster files.

## 3. Results

### 3.1. A Quality‐Controlled Atlas Resolves the Cellular Ecosystem of Treatment‐Naive Osteosarcoma

Across six tumors, 52,489 barcodes entered quality control, 43,339 passed core quality‐control filters, and 39,063 high‐quality singlets were retained (74.4% of input cells). Patient‐specific singlet retention ranged from 70.5% to 78.6%, supporting broadly comparable data quality without imposing an identical threshold on all samples (Figure S1; Table S1). SCTransform followed by Harmony generated a shared manifold containing 29 transcriptional clusters with extensive patient mixing, although several clusters retained patient‐associated abundance differences (Figure 1A,B; Figure S2).

**Figure 1:**
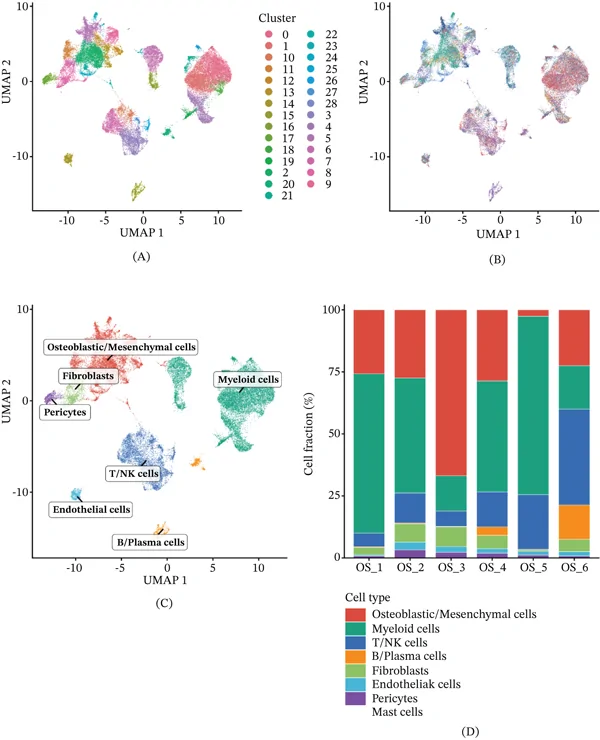
Integrated single‐cell landscape of human osteosarcoma. (A) Harmony‐integrated UMAP of 39,063 quality‐controlled cells colored by global cluster. (B) The same embedding colored by patient, showing cross‐patient mixing after integration. (C) Reviewed major‐cell‐type annotations displayed on the integrated UMAP; labels mark lineage centroids. (D) Relative abundance of major cell types in each patient. Colors are consistent between panels C and D.

Conservative marker‐guided review assigned 17,023 myeloid cells, 11,189 osteoblastic/mesenchymal cells, 6514 T/NK cells, 1836 fibroblasts, 1143 B/plasma cells, 711 endothelial cells, and 647 pericytes (Figure 1C,D). The abundance of these lineages varied substantially among tumors. This composition is consistent with the increasingly recognized view of osteosarcoma as a multicellular ecosystem rather than a uniform mass of malignant osteoblasts [28, 29]. Detailed annotation further resolved immune and stromal states, but these were retained as descriptive phenotypes because transcriptional boundaries were not always discrete (Figure S3).

### 3.2. Expression‐Derived CNV Patterns Support a Large Malignant Osteoblastic Compartment

Transcriptome‐derived CNV burden was concentrated in osteoblastic/mesenchymal cells, whereas immune and endothelial reference lineages had comparatively low scores (Figure 2A–C). Applying the reference‐cell 95th‐percentile CNV threshold (0.02019) together with osteoblastic lineage identity yielded 10,456 CNV‐supported malignant osteoblastic cells. Broad chromosome‐level deviations differed among malignant subclusters (Figure 2D). Among the observation groups, 1240 of 1836 fibroblasts (67.5%) and 157 of 647 pericytes (24.3%) exceeded the same CNV‐score threshold. These cells retained stromal marker profiles and were not reassigned as malignant because the classification rule required osteoblastic lineage identity and because expression‐derived CNV elevation alone is not definitive evidence of malignancy (Table S1).

**Figure 2:**
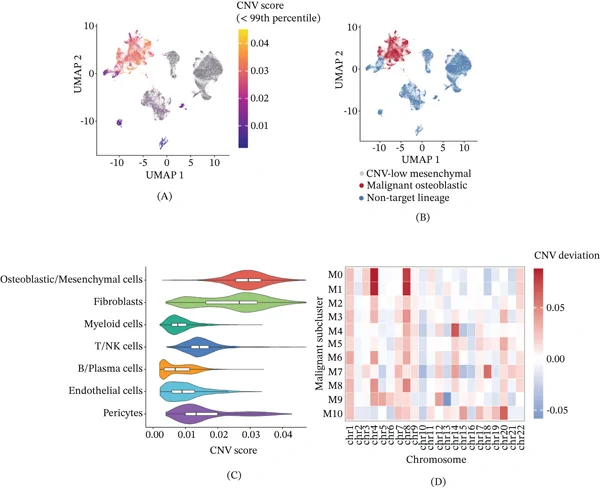
CNV‐supported identification of malignant osteoblastic cells. (A) Transcriptome‐derived CNV burden projected onto the global UMAP; values are capped at the 99th percentile for visualization. (B) Classification requiring both osteoblastic/mesenchymal lineage identity and a CNV score above the reference‐cell 95th percentile (0.02019). (C) Distribution of CNV scores across major lineages. (D) Mean chromosome‐level CNV deviation across malignant subclusters. Fibroblasts and pericytes were observation groups and were not reassigned on CNV score alone. RNA‐derived values are not direct DNA measurements.

These results fit the DNA‐level picture of osteosarcoma, in which copy‐number evolution and structural instability can generate multiple coexisting clones [3, 5]. They do not, however, establish the exact genotype of any cell. We therefore use “CNV‐supported malignant” throughout, reserving definitive copy‐number calls for DNA‐based profiling.

### 3.3. Malignant‐Cell Reclustering Identifies a Recurrent Proliferative/Mitotic M6 State

Independent reclustering of CNV‐supported malignant cells produced 11 subclusters (M0–M10; Figure 3A). M6 had the highest original six‐program composite score (0.942), followed by M8 (0.761), and was designated the M6 proliferative/mitotic state (Figure 3B–D). M6 contained 725 cells, was detected in all six patients, and represented 4.9%–18.6% of each patient′s malignant compartment and 6.9% of all malignant cells (Figure S4; Table S1). In leave‐one‐program‐out analyses, M6 ranked first in six of eight scenarios, ranked second when G2M checkpoint was removed, and ranked fifth when both G2M checkpoint and E2F targets were excluded (Table S2). Thus, M6 is recurrent across the six tumors, but its leading composite rank depends substantially on mitotic programs and should not be interpreted as a prospectively established clinical risk category.

**Figure 3:**
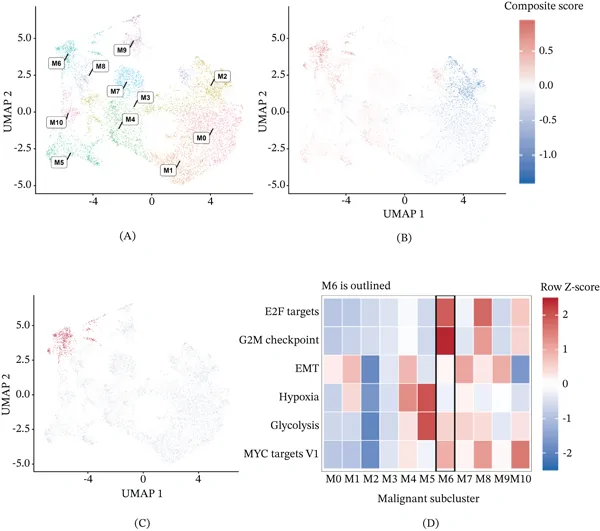
Malignant‐cell heterogeneity and definition of the M6 proliferative/mitotic state. (A) UMAP of 10,456 CNV‐supported malignant osteoblastic cells colored by subcluster. (B) Original composite score based on standardized E2F targets, G2M checkpoint, epithelial‐mesenchymal transition, hypoxia, glycolysis, and MYC targets V1 scores. (C) M6 highlighted against other malignant cells. (D) Row‐standardized program scores; M6 is outlined. Leave‐out sensitivity results are provided in Table S2.

The M6 designation captures a transcriptional state rather than a stable histological subtype. Its recurrence across all six specimens is supportive but does not establish broad prevalence across osteosarcoma, particularly given the small discovery cohort. Recent single‐cell studies likewise show that osteosarcoma malignant populations occupy multiple differentiation, stress, and proliferative states that can shift with treatment and anatomical context [8–10].

### 3.4. The M6 Program Is Organized Around Mitosis and Chromosome Segregation

Patient‐aware pseudobulk testing used 12 paired libraries—M6 and other malignant cells from each of six patients—with patient identity included in the edgeR design. The reanalysis exactly reproduced the original log2 fold‐changes across 14,292 tested genes (Pearson *r* = 1.000). It identified 2837 genes at FDR < 0.05, including 1066 higher and 1771 lower in M6. High‐confidence upregulated genes included UBE2C, CENPF, TOP2A, CCNB1, CDKN3, ARL6IP1, CDC20, KPNA2, HMGB2, and UBE2S (Figure 4A,B). Hallmark enrichment was strongest for G2M checkpoint (NES = 3.38), E2F targets (NES = 3.21), and mitotic spindle (NES = 3.03), whereas inflammatory response, TNF‐alpha signaling through NF‐kappaB, and selected differentiation‐associated programs were negatively enriched (Figure 4C). Gene Ontology analysis highlighted chromosome segregation, mitotic nuclear division, spindle assembly, and cytokinesis (Figure S5A).

**Figure 4:**
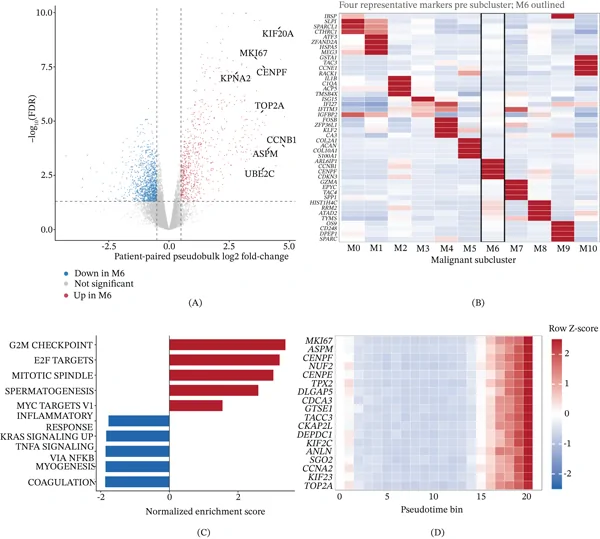
Molecular features of the M6 proliferative/mitotic state. (A) Patient‐paired pseudobulk differential expression comparing M6 with other malignant cells. (B) Row‐standardized expression of representative subcluster markers; M6 is outlined. (C) Hallmark enrichment in M6. (D) Descriptive candidate‐gene expression across 20 pseudotime bins along the post hoc selected M2‐M8‐M6 lineage. The trajectory is exploratory and is not evidence of temporal progression.

Slingshot inferred six exploratory lineages from M2, which was chosen as the starting cluster because it had the lowest original six‐program composite score; M6 was not imposed as an endpoint. The lineage containing M6 was selected post hoc and followed M2‐M8‐M6, with M6 at the endpoint (Figure S5B; Table S6). Candidate genes increased across the displayed pseudotime bins (Figure 4D; Figure S5C), but this pattern is partly conditional on selection of the M6‐containing lineage. It is therefore presented only as descriptive transcriptional continuity, not as independent evidence that other malignant cells progress toward M6 in vivo.

### 3.5. The M6‐Derived Program Is Associated With Metastatic Occurrence in GSE21257

Fifty single‐cell‐derived candidates mapped to the GSE21257 platform. All 19 patients classified as nonmetastatic had at least 60 months of recorded follow‐up. The weighted M6‐derived module score was higher in pretreatment tumors from patients who developed metastasis within 5 years (Wilcoxon *p* = 0.0147) and had an apparent AUC of 0.704 (95% CI 0.562–0.847; Figure 5A). The expression heat map showed coordinated elevation of multiple mitotic candidates in a subset of metastatic tumors rather than complete separation of clinical groups (Figure 5B). This result represents a cross‐cohort association and cannot establish that the bulk signal originated specifically from M6 cells.

**Figure 5:**
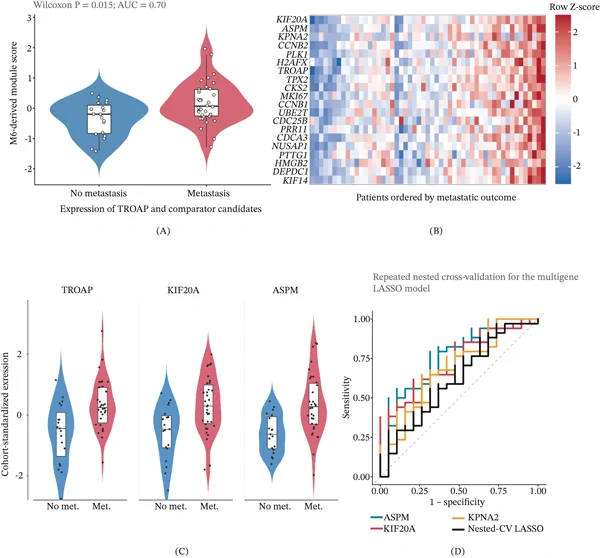
Cross‐cohort bulk association and candidate evaluation in GSE21257. (A) M6‐derived module scores in nonmetastatic and metastatic tumors; all nonmetastatic patients had at least 60 months of follow‐up. The displayed AUC is apparent. (B) Row‐standardized expression of the 50 mapped module genes. (C) Expression of TROAP and comparator candidates KIF20A and ASPM. (D) ROC curves for the nested‐cross‐validation LASSO model and descriptive single‐gene models. Bulk data cannot establish that the module originates specifically from M6 cells.

At the single‐gene level, *ASPM*, *KIF20A*, and *TROAP* were higher in metastatic tumors, with apparent AUCs of 0.788, 0.757, and 0.746, respectively (Figure 5C,D). Their nominal logistic‐regression *p* values were 0.003, 0.004, and 0.0035. Only *ASPM* remained below a 5% genome‐wide FDR; *TROAP* was borderline after adjustment (FDR = 0.051), and *KIF20A* had FDR = 0.071 (Figure S6B). The pattern supports clinical association while illustrating the uncertainty imposed by 53 samples.

Repeated nested five‐fold cross‐validation produced an AUC of 0.659 (95% CI 0.505–0.814) for the multigene LASSO model (Figure 5D). ASPM and TROAP were among the more frequently retained candidates across nested resampling, although selection remained incomplete (Figure S7A). The modest AUC and wide confidence interval do not support a clinical‐prediction claim; the model was used only to inform candidate prioritization.

### 3.6. Transparent Multidomain Evidence Supports TROAP for Functional Follow‐Up

TROAP was detected in 80.4% of M6 cells compared with 11.7% of other malignant cells, with an average cell‐level log2 fold‐change of 1.18. Its increase persisted after patient aggregation (pseudobulk log2 fold − change = 3.16; FDR = 2.73 × 10^−5^). In GSE21257, TROAP was higher in metastatic tumors (log2 fold − change = 0.50; nominal *p* = 4.97 × 10^−4^; FDR = 0.051) and showed a descriptive univariable association with metastatic occurrence (OR per standard − deviation increase = 3.52, 95% CI 1.51–8.18; *p* = 0.0035). TROAP was selected in 53% of repeated nested‐cross‐validation fits and 33% of bootstrap fits.

TROAP was not the strongest candidate on every statistical measure. KIF20A ranked first and ASPM ranked second in the original integrated ranking, whereas TROAP ranked seventh. Under the four transparent revision weighting scenarios, TROAP ranked 5th, 11th, 5th, and 3rd, respectively (Table S5). It was retained for functional follow‐up as one of several Tier A candidates because its single‐cell localization, paired‐pseudobulk evidence, directionally concordant bulk association, selection stability, biological interpretability, and limited osteosarcoma‐specific characterization formed a coherent experimental rationale. The choice should therefore be understood as biologically motivated prioritization, not as proof that TROAP was the unique statistical optimum.

In GSE21257, the M6‐derived module was associated with metastatic occurrence before adjustment (OR = 2.633 per standard‐deviation increase, 95% CI 1.235–5.614; *p* = 0.0122). After joint modeling with the macrophage score, the module association was attenuated and no longer statistically significant (OR = 1.646, 95% CI 0.649–4.170; *p* = 0.2937), whereas the macrophage score remained inversely associated with metastasis (OR = 0.251, 95% CI 0.081–0.773; *p* = 0.0161; Figure 6A,B; Figure S8B). This analysis indicates that the bulk module association is sensitive to tumor composition and should not be interpreted as an independent clinical effect.

**Figure 6:**
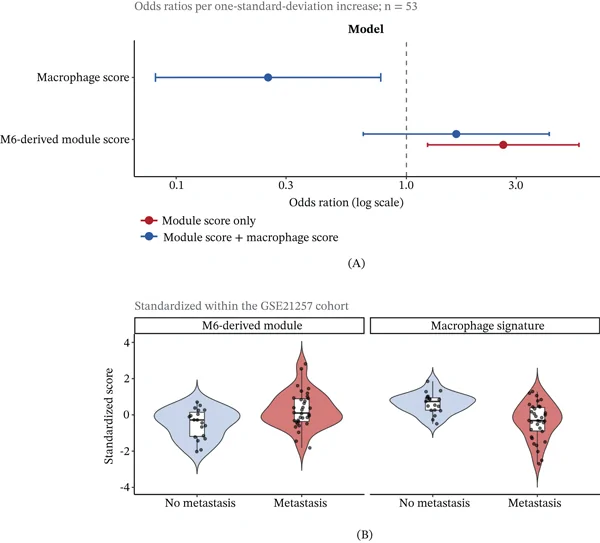
Microenvironment sensitivity of the M6‐derived bulk association. (A) Odds ratios per one‐standard‐deviation increase from the unadjusted module model and the joint module‐plus‐macrophage model. Error bars show 95% confidence intervals. (B) Distributions of standardized M6‐derived module and macrophage scores by metastatic status in GSE21257. The attenuation of the module estimate after macrophage adjustment indicates sensitivity to tumor composition.

### 3.7. TROAP Depletion Reduces Clonogenic Growth, Wound Closure, Migration, and Invasion

Two independent siRNAs reduced TROAP protein abundance in both HOS and U2OS cells relative to siNC (Figure 7A). TROAP depletion decreased colony formation in both lines (Figure 7B) and reduced overall wound closure at 24 h (Figure 7C). Migration and invasion through Transwell membranes were also reduced after either siRNA (Figure 7D). All quantified comparisons with siNC reached *p* < 0.001. Because the scratch assay was not adjusted for the reduction in proliferative or clonogenic capacity, its result cannot be attributed to migration alone.

**Figure 7:**
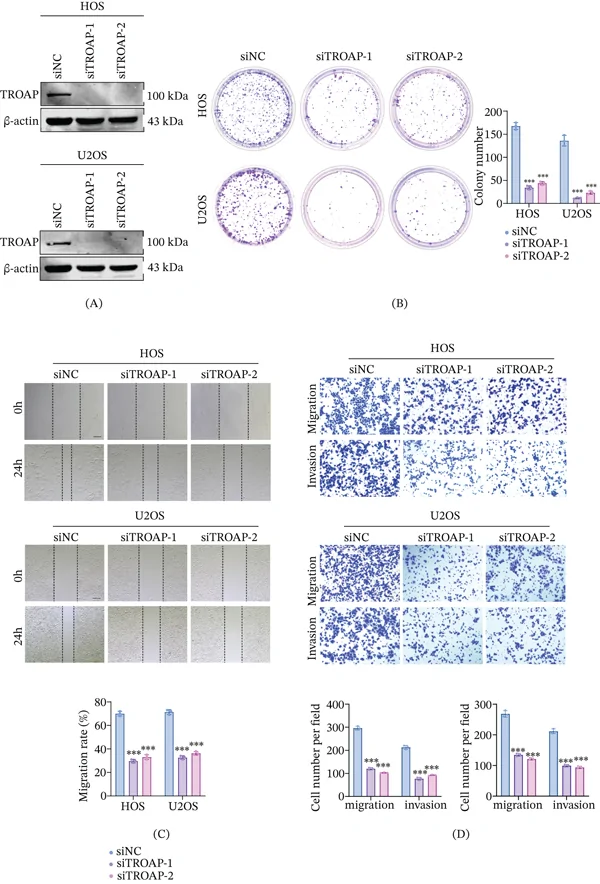
TROAP depletion reduces clonogenic growth, overall wound closure, migration, and invasion in osteosarcoma cells. (A) Immunoblot analysis of TROAP in HOS and U2OS cells transfected with siNC, siTROAP‐1, or siTROAP‐2; beta‐actin is the loading control. (B) Colony‐formation images and colony counts. (C) Scratch‐wound images at 0 and 24 h and overall wound closure. Because the assay was not proliferation‐matched, it is not interpreted as direct migration evidence. (D) Transwell migration and invasion images and cell counts. Data are mean + /−standard deviation from three independent biological experiments. One‐way ANOVA with Dunnett′s test compared each siTROAP group with siNC;  ^∗∗∗^ *p* < 0.001.

The agreement between two siRNAs and two cell lines reduces the likelihood that the observed phenotype reflects a single reagent or cellular background. Together with the transcriptomic evidence, the results support a functional contribution of TROAP to clonogenic growth and motility‐related phenotypes. They do not establish that TROAP drives the M6 state, directly mediates metastatic behavior in patients, or acts independently of its effects on proliferation.

## 4. Discussion

This study identifies a recurrent proliferative/mitotic malignant state in treatment‐naive osteosarcoma and evaluates its transcriptional program in an independent pretreatment bulk cohort. The main contribution is a traceable evidence chain: a CNV‐supported malignant subpopulation was detected in all six tumors, characterized with patient‐paired pseudobulk analysis, evaluated for association with metastatic occurrence, and used to nominate experimentally testable candidates. The additional sensitivity analyses also define the limits of that chain. M6 ranking depends substantially on G2M and E2F programs, the bulk association is attenuated after macrophage adjustment, and TROAP is one of several plausible candidates rather than a uniformly top‐ranked gene.

### 4.1. A Malignant State Can Be More Reproducible Than a Single Genomic Lesion

M6 may represent one such transcriptional convergence: its defining genes govern chromosome segregation, spindle function, G2/M progression, and cytokinesis. The program‐selection sensitivity analysis, however, shows that this identity is inseparable from its proliferative content. We therefore describe M6 as a proliferative/mitotic malignant state, not as an intrinsically high‐risk biological subtype. Whether this state has adverse clinical consequences must be evaluated separately from how it was transcriptionally defined.

The inference must remain bounded. RNA‐derived CNV separates broad malignant and nonmalignant expression patterns but cannot reconstruct the rearrangement architecture of osteosarcoma. The chromosome‐level heat map in Figure 2 is therefore supportive context, not proof that a particular gain or loss causes M6. Matched single‐cell DNA and RNA profiling would be required to determine whether M6 is anchored to a stable clone, emerges transiently across clones, or reflects both processes.

### 4.2. M6 Is a Coordinated Proliferative State Rather Than an MKI67‐Only Cluster

High proliferation can dominate unsupervised single‐cell clustering and create an apparently distinct population. M6 was represented in every patient and showed a broad mitotic network in paired pseudobulk analysis, but leave‐out analyses demonstrated that its top composite ranking was not maintained after joint removal of G2M checkpoint and E2F targets. This finding resolves the apparent circularity: the data support a coordinated proliferative/mitotic state, whereas the adverse‐risk interpretation arises only from subsequent bulk‐cohort association. Recurrence in six specimens is supportive but remains limited evidence of broad osteosarcoma prevalence.

This interpretation aligns with recent osteosarcoma atlases that describe proliferative osteoblastic subsets and treatment‐associated shifts in malignant state composition [8, 9]. However, our cross‐sectional pseudotime should not be read as evidence that M6 is a terminal evolutionary fate. Its most defensible use is to order transcriptionally related cells and visualize coordinated gene behavior. Longitudinal sampling, lineage tracing, or perturbation‐based state‐transition experiments are needed to establish directionality.

### 4.3. Cross‐Cohort Bulk Association Supports Relevance But Not a Deployable Predictor

The GSE21257 analysis was constrained to candidates derived from the single‐cell dataset. The higher M6‐derived module score in metastatic tumors indicates that this transcriptional program is detectable across an independent cohort, but bulk data cannot identify its cellular source. The nested‐cross‐validated AUC of 0.659 and wide confidence interval further show that the signal is not ready for clinical use. We therefore interpret the result as a cross‐cohort association with metastatic occurrence rather than cell‐state‐specific validation or a deployable predictor.

The attenuation of the M6‐derived module after macrophage adjustment is central to interpretation. GSE21257 originally associated macrophage infiltration with suppression of metastasis [16]. In our joint model, the macrophage score remained inversely associated with metastatic occurrence, whereas the module estimate weakened and crossed the null. A bulk biopsy integrates malignant‐state abundance with immune and stromal composition, and the two scores were moderately inversely correlated (Figure S8A). The adjusted result therefore does not negate the single‐cell state; it shows that the bulk association cannot be assigned independently to that state. Future cohorts should measure malignant and microenvironmental axes jointly.

### 4.4. Why TROAP Was Prioritized

TROAP was not the strongest gene on every metric. KIF20A and ASPM achieved higher overall statistical ranks, and ASPM showed a higher apparent AUC and stronger genome‐wide bulk evidence. TROAP nevertheless remained within the high‐priority group across most transparent weighting scenarios and showed marked M6 enrichment, patient‐paired pseudobulk support, directionally concordant bulk association, and moderate resampling stability. Functional follow‐up therefore tested one biologically coherent candidate from the shortlist; it did not confirm that TROAP was the unique or statistically dominant candidate.

The known biology of TROAP provides a plausible connection to the M6 phenotype. TROAP has been implicated in cell‐cycle control, spindle‐associated processes, proliferation, and motility in several cancer contexts [19–21]. In osteosarcoma cells, two independent siRNAs reduced clonogenic growth, wound closure, and Transwell migration and invasion. These results support a functional contribution to malignant phenotypes but do not show that TROAP creates the M6 program or mediates its bulk association with metastasis. Reduced proliferation may also contribute to the wound‐closure and Transwell phenotypes.

That possibility suggests testable mechanisms. Because M6 is enriched for spindle and cytokinesis genes, TROAP depletion may alter mitotic fidelity, cell‐cycle progression, or survival after chromosome‐segregation stress. The motility phenotype may be secondary to reduced proliferation, a direct cytoskeletal effect, or both. Time‐matched proliferation controls, cell‐cycle profiling, live‐cell mitotic imaging, rescue with siRNA‐resistant TROAP, and pathway‐level perturbation will be needed to distinguish these models. The present experiments validate direction and phenotype; they do not yet resolve mechanism.

### 4.5. Translational Implications

The most immediate implication is prioritization, not treatment selection. TROAP may be useful as a marker of a proliferative malignant compartment, as a perturbation target for mechanistic studies, or as one component of a multiparametric risk panel. A therapy directed at a cell‐cycle‐linked target also raises predictable concerns: proliferating normal tissues may share the dependency, and intratumoral state plasticity may allow escape. Spatial data increasingly show that malignant states and immune neighborhoods are organized together in osteosarcoma [11, 30]. It will therefore be important to determine whether TROAP‐high cells occupy particular vascular, hypoxic, necrotic, or immune‐excluded niches and whether those niches influence drug response.

The experimental strategy can also be generalized. Rather than train a complex model on a small bulk cohort and retrospectively seek biological meaning, single‐cell analysis first defines a cellular program, patient‐pseudobulk analysis tests its recurrence, bulk data assess clinical direction, and functional assays test a selected component. This ordering reduces—but does not eliminate—overfitting and makes negative or equivocal evidence visible. The borderline bulk FDR for *TROAP* and modest cross‐validated model performance are therefore not hidden weaknesses; they define the level of evidence and the next validation step.

### 4.6. Limitations

Several limitations constrain interpretation. GSE162454 contains only six tumors, with no matched normal bone or longitudinal samples, so detection of M6 in all six specimens cannot establish population‐wide prevalence. RNA‐derived CNV is an indirect malignancy measure; notably, subsets of fibroblasts and pericytes also showed elevated scores and were retained under their stromal identities. M6 classification depends on selected programs, and its rank decreased after removal of proliferation‐related programs. GSE21257 is a small retrospective microarray cohort; bulk measurements cannot localize the signal to M6; the nested‐cross‐validated performance was modest; and the module association was not independently significant after macrophage adjustment. These constraints support cautious association rather than causal or clinical‐prediction claims.

The functional experiments used established cell lines and short‐term RNA interference. Although two siRNAs and two cellular backgrounds yielded consistent results, no rescue experiment, proliferation‐matched motility analysis, cell‐cycle profiling, patient‐derived model, or in vivo metastasis study was performed. In particular, reduced proliferation or survival may contribute to lower wound closure and Transwell counts. The current experiments therefore establish directionally consistent phenotypic effects of TROAP depletion but do not define mechanism or prove a direct migration‐specific role.

## 5. Conclusion

Integrating single‐cell discovery with patient‐aware replication, cross‐cohort bulk association, and focused perturbation identified a recurrent proliferative/mitotic malignant state in six human osteosarcomas. Its derived program was associated with metastatic occurrence in GSE21257 before, but not after, macrophage adjustment. TROAP was selected as one of several high‐priority candidates, and its depletion reduced clonogenic growth, overall wound closure, and Transwell migration and invasion in HOS and U2OS cells. These findings support TROAP as a functional contributor to osteosarcoma cell phenotypes while leaving its mechanistic and clinical roles to be tested in larger and more direct models.

## Author Contributions

P.J.: conceptualization, methodology, formal analysis, visualization, and writing—original draft. X.C.: conceptualization, methodology, formal analysis, visualization, and writing—original draft. H.W.: investigation, data curation, validation, and writing—review and editing. Z.S:. investigation, data curation, validation, and writing—review and editing. M.L.: investigation, data curation, validation, and writing—review and editing. Y.B.: investigation, data curation, validation, and writing—review and editing. W.Z.: supervision, project administration, methodology, and writing—review and editing. D.W.: supervision, project administration, conceptualization, and writing—review and editing. All authors contributed to the article and approved the submitted version. P.J. and X.C. authors contributed equally to this work.

## Funding

No funding was received for this manuscript.

## Disclosure

The authors reviewed and edited the manuscript in full and take complete responsibility for its scientific content, accuracy, interpretations, and conclusions.

## Ethics Statement

This study reanalyzed publicly available, de‐identified human transcriptomic datasets. The original studies reported their respective ethical approvals and consent procedures. No new human participants or animals were enrolled. Experiments were performed in established human osteosarcoma cell lines. Because this study used only publicly available, de‐identified data, no additional institutional ethical approval or informed consent was required.

## Conflicts of Interest

The authors declare that the research was conducted in the absence of any commercial or financial relationships that could be construed as a potential conflict of interest.

## Supporting information


**Supporting Information** Additional supporting information can be found online in the Supporting Information section. Figure S1: Single‐cell quality control. (A) Patient‐specific distributions of detected genes per cell. Black dashed and red dotted lines indicate lower and upper sample‐specific thresholds. (B) Mitochondrial‐read distributions with sample‐specific thresholds marked in red. (C) Fraction of cells retained after core quality control and sample‐aware doublet removal. Figure S2: Global‐cluster diagnostics. (A) Fractional abundance of each global cluster by patient. (B) Number of cells assigned to each global cluster. Figure S3: Detailed cell‐state landscape. (A) Integrated UMAP colored by 25 detailed cell states. State codes are placed at cluster centroids. (B) Relative abundance of detailed cell states in each patient, with the complete code‐to‐state mapping shown in the legend. Detailed states are descriptive transcriptional assignments. Figure S4: Malignant‐state marker and composition analyses. (A) Full row‐standardized heat map of representative markers across malignant subclusters. (B) Malignant‐subcluster composition by patient. (C) Fraction of malignant cells assigned to the M6 proliferative/mitotic state in each patient. Figure S5: Functional enrichment and exploratory transcriptional ordering. (A) Nonredundant Gene Ontology terms enriched among M6 markers. (B) Exploratory Slingshot pseudotime projected onto the malignant‐cell UMAP. M2 was supplied as the start; no endpoint was imposed, and the M2‐M8‐M6 lineage was selected post hoc. (C) Candidate‐gene dynamics across 20 equal‐frequency pseudotime bins. This analysis is descriptive and does not establish temporal progression. Figure S6: Bulk‐cohort quality control and candidate associations. (A) Principal‐component analysis of GSE21257 profiles colored by metastatic outcome. (B) Bulk associations of mapped single‐cell candidate genes with metastatic outcome. Selected targets are labeled. Figure S7: Model stability and integrated target evidence. (A) Nested‐cross‐validation and bootstrap selection frequencies for the 20 most frequently selected LASSO candidates. (B) Scaled evidence matrix used for consensus target ranking across single‐cell specificity, bulk effect, bulk statistical evidence, single‐gene discrimination, LASSO stability, adjusted robustness, and consensus score. Figure S8: Microenvironment sensitivity and exploratory survival. (A) Relationship between macrophage and M6‐derived module scores, stratified by metastatic outcome. (B) Joint logistic model: module OR = 1.646 (95% CI 0.649–4.170; *p* = 0.2937) and macrophage OR = 0.251 (95% CI 0.081–0.773; *p* = 0.0161). (C) Exploratory Kaplan–Meier curves after median dichotomization of the module score. Survival results were not used for target selection. Table S1: Patient‐level cell flow and malignant‐state retention. Counts at each processing step and M6 proportions for each patient, together with the lineage‐level CNV‐score audit. Table S2: Raw program scores and original composite score. Raw program scores and composite scores by malignant subcluster, with leave‐one‐program‐out and joint G2M/E2F exclusion analyses. Table S3: Complete 50‐gene module definition and weights. Gene identities, positive directions, normalized weights, and corresponding pseudobulk and bulk differential‐expression statistics. Table S4: GSE21257 sample‐level module scores. Sample identifiers, metastatic outcome, recorded follow‐up, and M6‐derived module and macrophage scores. Table S5: Candidate ranks under all weighting scenarios. Candidate ranks under the four revision weighting scenarios and the original integrated ranking. Table S6: Statistical and trajectory audit summary. Summary of the patient‐paired pseudobulk design, reproducibility checks, module‐score reconstruction, follow‐up audit, and Slingshot settings.

## Data Availability

The datasets analyzed in this study are publicly available in the NCBI Gene Expression Omnibus under accession numbers GSE162454 and GSE21257. The analysis code and processed source‐data tables supporting the findings of this study are available from the primary corresponding author, Dexin Wang (wdx19860318@163.com), upon reasonable request.
