# Supplementary material for: Integrative Single‐Cell and Bulk Transcriptomics Identifies a Proliferative Malignant State and Supports a Functional Contribution of TROAP in Osteosarcoma
Source: Int J Genomics. 2026 Oct 14;2026:1407947. doi: 10.1155/ijog/1407947 (PMC13669654; doi:10.1155/ijog/1407947)
Supplement: Supplementary file 1 [file IJOG-2026-1407947-s001.docx]

**Supplementary Material**

**Supplementary Figures**

**Supplementary Figure S1. Single-cell quality control**

(A) Patient-specific distributions of detected genes per cell. Black dashed and red dotted lines indicate lower and upper sample-specific thresholds. (B) Mitochondrial-read distributions with sample-specific thresholds marked in red. (C) Fraction of cells retained after core quality control and sample-aware doublet removal.

**Supplementary Figure S2. Global-cluster diagnostics**

(A) Fractional abundance of each global cluster by patient. (B) Number of cells assigned to each global cluster.

**Supplementary Figure S3. Detailed cell-state landscape**

(A) Integrated UMAP colored by 25 detailed cell states. State codes are placed at cluster centroids. (B) Relative abundance of detailed cell states in each patient, with the complete code-to-state mapping shown in the legend. Detailed states are descriptive transcriptional assignments.

**Supplementary Figure S4. Malignant-state marker and composition analyses**

(A) Full row-standardized heatmap of representative markers across malignant subclusters. (B) Malignant-subcluster composition by patient. (C) Fraction of malignant cells assigned to the M6 proliferative/mitotic state in each patient.

**Supplementary Figure S5. Functional enrichment and exploratory transcriptional ordering**

(A) Nonredundant Gene Ontology terms enriched among M6 markers. (B) Exploratory Slingshot pseudotime projected onto the malignant-cell UMAP. M2 was supplied as the start; no endpoint was imposed, and the M2-M8-M6 lineage was selected post hoc. (C) Candidate-gene dynamics across 20 equal-frequency pseudotime bins. This analysis is descriptive and does not establish temporal progression.

**Supplementary Figure S6. Bulk-cohort quality control and candidate associations**

(A) Principal-component analysis of GSE21257 profiles colored by metastatic outcome. (B) Bulk associations of mapped single-cell candidate genes with metastatic outcome. Selected targets are labeled.

**Supplementary Figure S7. Model stability and integrated target evidence**

(A) Nested-cross-validation and bootstrap selection frequencies for the 20 most frequently selected LASSO candidates. (B) Scaled evidence matrix used for consensus target ranking across single-cell specificity, bulk effect, bulk statistical evidence, single-gene discrimination, LASSO stability, adjusted robustness, and consensus score.

**Supplementary Figure S8. Microenvironment sensitivity and exploratory survival**

(A) Relationship between macrophage and M6-derived module scores, stratified by metastatic outcome. Lines show group-specific linear fits. (B) Joint logistic model: module OR = 1.646 (95% CI 0.649-4.170; P = 0.2937) and macrophage OR = 0.251 (95% CI 0.081-0.773; P = 0.0161). (C) Exploratory Kaplan-Meier curves after median dichotomization of the module score. Survival results were not used for target selection.

**Supplementary Tables**

**Supplementary Table S1. Patient-level cell flow and malignant-state retention**

| **Patient** | **Input** | **Core QC** | **Singlets** | **CNV-supported malignant** | **M6** | **M6 fraction** |
| --- | --- | --- | --- | --- | --- | --- |
| OS_1 | 8894 | 7606 | 6898 | 1481 | 114 | 7.7% |
| OS_2 | 8331 | 7223 | 6547 | 1692 | 153 | 9.0% |
| OS_3 | 9881 | 8249 | 7260 | 4803 | 237 | 4.9% |
| OS_4 | 4768 | 3817 | 3545 | 925 | 77 | 8.3% |
| OS_5 | 10926 | 8827 | 7978 | 177 | 33 | 18.6% |
| OS_6 | 9689 | 7617 | 6835 | 1378 | 111 | 8.1% |

**Supplementary Table S1 continued. CNV-score audit by lineage**

| **Lineage** | **Scored cells** | **Above 0.02019** | **Above threshold (%)** | **Median CNV score** |
| --- | --- | --- | --- | --- |
| B/Plasma cells | 1143 | 5 | 0.4 | 0.0068 |
| Endothelial cells | 711 | 46 | 6.5 | 0.0089 |
| Fibroblasts | 1836 | 1240 | 67.5 | 0.0267 |
| Myeloid cells | 1500 | 25 | 1.7 | 0.0076 |
| Osteoblastic/Mesenchymal cells | 11189 | 10456 | 93.4 | 0.0294 |
| Pericytes | 647 | 157 | 24.3 | 0.0133 |
| T/NK cells | 1500 | 167 | 11.1 | 0.0143 |

**Supplementary Table S2. Raw program scores and original composite score**

| **Cluster** | **Cells** | **Patients** | **E2F** | **G2M** | **EMT** | **Hypoxia** | **Glycolysis** | **MYC** | **Composite** |
| --- | --- | --- | --- | --- | --- | --- | --- | --- | --- |
| M0 | 2120 | 6 | 0.066 | 0.054 | 0.147 | 0.081 | 0.080 | 0.262 | -0.619 |
| M1 | 1723 | 6 | 0.068 | 0.060 | 0.164 | 0.106 | 0.080 | 0.260 | -0.301 |
| M10 | 329 | 5 | 0.133 | 0.110 | 0.090 | 0.086 | 0.095 | 0.375 | 0.155 |
| M2 | 1157 | 6 | 0.079 | 0.066 | 0.083 | 0.061 | 0.060 | 0.218 | -1.405 |
| M3 | 973 | 6 | 0.085 | 0.073 | 0.128 | 0.085 | 0.080 | 0.283 | -0.454 |
| M4 | 931 | 6 | 0.100 | 0.086 | 0.166 | 0.124 | 0.094 | 0.316 | 0.433 |
| M5 | 762 | 6 | 0.078 | 0.063 | 0.127 | 0.139 | 0.121 | 0.292 | 0.361 |
| M6 | 725 | 6 | 0.188 | 0.191 | 0.144 | 0.091 | 0.097 | 0.347 | 0.942 |
| M7 | 671 | 4 | 0.095 | 0.072 | 0.172 | 0.100 | 0.097 | 0.311 | 0.217 |
| M8 | 586 | 6 | 0.182 | 0.139 | 0.148 | 0.091 | 0.096 | 0.358 | 0.761 |
| M9 | 479 | 5 | 0.078 | 0.065 | 0.169 | 0.095 | 0.084 | 0.308 | -0.090 |

**Supplementary Table S2 continued. M6 program-selection sensitivity**

| **Scenario** | **Excluded program(s)** | **M6 composite** | **M6 rank** | **Top cluster** |
| --- | --- | --- | --- | --- |
| All_six_programs |  | 0.942 | 1 | M6 |
| Leave_out_E2f_Targets | HALLMARK_E2F_TARGETS | 0.749 | 1 | M6 |
| Leave_out_G2m_Checkpoint | HALLMARK_G2M_CHECKPOINT | 0.647 | 2 | M8 |
| Leave_out_Epithelial_Mesenchymal_Transition | HALLMARK_EPITHELIAL_MESENCHYMAL_TRANSITION | 1.103 | 1 | M6 |
| Leave_out_Hypoxia | HALLMARK_HYPOXIA | 1.184 | 1 | M6 |
| Leave_out_Glycolysis | HALLMARK_GLYCOLYSIS | 1.029 | 1 | M6 |
| Leave_out_Myc | HALLMARK_MYC_TARGETS_V1 | 0.940 | 1 | M6 |
| Exclude_G2M_and_E2F | HALLMARK_E2F_TARGETS;HALLMARK_G2M_CHECKPOINT | 0.332 | 5 | M5 |

**Supplementary Table S3. Complete 50-gene module definition and weights**

| **Gene** | **Direction** | **Weight** | **M6 pseudobulk logFC** | **M6 FDR** | **Bulk logFC** | **Bulk FDR** |
| --- | --- | --- | --- | --- | --- | --- |
| MKI67 | positive | 0.024 | 3.648 | 1.44e-08 | 0.268 | 0.149 |
| CENPF | positive | 0.024 | 3.874 | 2.76e-08 | 0.356 | 0.312 |
| PTTG1 | positive | 0.024 | 3.206 | 1.08e-10 | 0.203 | 0.610 |
| CCNB2 | positive | 0.024 | 3.964 | 3.55e-08 | 0.593 | 0.116 |
| NEK2 | positive | 0.023 | 4.638 | 1.44e-08 | 0.201 | 0.270 |
| HMGB2 | positive | 0.023 | 2.673 | 1.08e-10 | 0.213 | 0.493 |
| TOP2A | positive | 0.022 | 3.810 | 4.17e-06 | 0.227 | 0.571 |
| CKAP2 | positive | 0.022 | 3.233 | 9.35e-08 | 0.131 | 0.529 |
| CENPE | positive | 0.022 | 4.058 | 1.71e-06 | 0.344 | 0.321 |
| NUSAP1 | positive | 0.022 | 3.372 | 3.68e-06 | 0.418 | 0.191 |
| KIF20A | positive | 0.022 | 4.628 | 4.44e-10 | 0.595 | 0.071 |
| HMGB3 | positive | 0.021 | 1.963 | 4.44e-10 | 0.178 | 0.484 |
| KPNA2 | positive | 0.021 | 2.853 | 5.30e-08 | 1.016 | 0.137 |
| PLK1 | positive | 0.021 | 4.794 | 6.22e-06 | 0.310 | 0.082 |
| TPX2 | positive | 0.021 | 3.586 | 1.37e-05 | 0.564 | 0.070 |
| CKAP5 | positive | 0.021 | 2.419 | 1.25e-08 | 0.139 | 0.617 |
| PRR11 | positive | 0.021 | 3.777 | 2.31e-10 | 0.245 | 0.304 |
| DEPDC1 | positive | 0.021 | 3.750 | 1.64e-05 | 0.448 | 0.208 |
| BIRC5 | positive | 0.021 | 3.091 | 1.62e-05 | 0.115 | 0.541 |
| CKS2 | positive | 0.020 | 2.450 | 1.07e-07 | 0.695 | 0.104 |
| HMMR | positive | 0.020 | 3.981 | 1.60e-05 | 0.383 | 0.267 |
| CDKN3 | positive | 0.020 | 3.587 | 6.35e-05 | 0.118 | 0.776 |
| CENPA | positive | 0.020 | 4.326 | 4.86e-05 | 0.303 | 0.443 |
| CDC25B | positive | 0.020 | 3.030 | 1.31e-06 | 0.372 | 0.218 |
| CCNB1 | positive | 0.020 | 4.794 | 1.57e-04 | 0.606 | 0.126 |
| H2AFX | positive | 0.020 | 1.614 | 1.25e-08 | 0.425 | 0.150 |
| GTSE1 | positive | 0.020 | 3.698 | 2.58e-05 | 0.265 | 0.191 |
| PHF19 | positive | 0.019 | 2.092 | 1.54e-07 | 0.053 | 0.873 |
| KIF11 | positive | 0.019 | 3.020 | 2.27e-08 | 0.177 | 0.451 |
| ASPM | positive | 0.019 | 4.172 | 1.65e-04 | 0.902 | 0.042 |
| PRC1 | positive | 0.019 | 3.245 | 8.35e-06 | 0.310 | 0.421 |
| TROAP | positive | 0.019 | 3.155 | 2.73e-05 | 0.500 | 0.051 |
| KIF2C | positive | 0.019 | 3.837 | 4.17e-05 | 0.200 | 0.596 |
| RANGAP1 | positive | 0.019 | 2.155 | 5.32e-08 | 0.076 | 0.770 |
| ECT2 | positive | 0.018 | 2.828 | 8.82e-08 | -0.036 | 0.890 |
| DTYMK | positive | 0.018 | 1.474 | 8.82e-08 | 0.080 | 0.808 |
| BUB3 | positive | 0.018 | 1.483 | 3.69e-07 | 0.646 | 0.171 |
| KIF14 | positive | 0.018 | 4.027 | 5.49e-06 | 0.414 | 0.105 |
| UBE2S | positive | 0.018 | 2.463 | 4.44e-10 | 0.284 | 0.393 |
| CCNA2 | positive | 0.018 | 3.655 | 2.45e-04 | 0.372 | 0.198 |
| ANP32E | positive | 0.018 | 1.550 | 4.26e-08 | 0.022 | 0.526 |
| UBE2T | positive | 0.018 | 1.869 | 6.99e-06 | 0.566 | 0.107 |
| DBF4 | positive | 0.018 | 2.148 | 5.22e-07 | 0.023 | 0.531 |
| RACGAP1 | positive | 0.018 | 3.303 | 5.77e-05 | 0.100 | 0.396 |
| LBR | positive | 0.018 | 2.101 | 3.46e-08 | -0.081 | 0.799 |
| CDCA3 | positive | 0.018 | 3.791 | 2.32e-04 | 0.654 | 0.079 |
| LMNB1 | positive | 0.018 | 1.686 | 1.02e-07 | 0.351 | 0.269 |
| KIF22 | positive | 0.018 | 2.293 | 7.89e-06 | 0.230 | 0.293 |
| ANLN | positive | 0.018 | 3.097 | 5.53e-05 | 0.559 | 0.150 |
| TACC3 | positive | 0.018 | 3.382 | 2.21e-04 | 0.587 | 0.105 |

**Supplementary Table S4. GSE21257 sample-level module scores**

| **GEO sample** | **Patient** | **Outcome group** | **Recorded follow-up (months)** | **M6 module score** | **Macrophage score** |
| --- | --- | --- | --- | --- | --- |
| GSM530667 | 00043-05 | Metastasis | 27 | 0.1939 | 0.3771 |
| GSM530899 | 00745-04 | Metastasis | 21 | 0.0525 | -0.7773 |
| GSM531283 | 00917-04 | Metastasis | 46 | 1.7765 | -1.2724 |
| GSM531284 | 07677-05 | Metastasis | 28 | 0.9161 | -0.2726 |
| GSM531285 | 09541-05 | Metastasis | 11 | 0.2850 | -0.3467 |
| GSM531286 | 09799-04 | Metastasis | 37 | -0.0952 | -1.4398 |
| GSM531287 | 14971-03 | Metastasis | 45 | -0.4148 | 0.4036 |
| GSM531288 | 16530-03 | Metastasis | 13 | -0.3401 | -0.0691 |
| GSM531289 | 17022-03 | Metastasis | 33 | 0.2746 | -0.6897 |
| GSM531290 | 19264-05 | Metastasis | 25 | 1.9675 | -1.7255 |
| GSM531291 | 1988-92P | Metastasis | 18 | -0.2617 | -0.2651 |
| GSM531292 | 20014-05 | Metastasis | 30 | -0.6732 | 0.3709 |
| GSM531293 | 21150-03 | Metastasis | 35 | -0.3402 | 0.0886 |
| GSM531294 | 24939-05 | Metastasis | 4 | 0.4445 | -0.6271 |
| GSM531295 | 26814-04 | Metastasis | 27 | 0.5280 | -1.1072 |
| GSM531296 | 35215-05 | Metastasis | 26 | -0.0368 | 0.4907 |
| GSM531297 | 35855-05 | Metastasis | 18 | -0.2098 | -0.2954 |
| GSM531298 | 4474-05P | Metastasis | 189 | -1.0128 | -2.1410 |
| GSM531299 | IOR-001 | Metastasis | 36 | 0.6327 | -1.0463 |
| GSM531300 | IOR-004 | Metastasis | 123 | 0.0876 | -0.4438 |
| GSM531301 | IOR-008 | Metastasis | 110 | 0.7078 | -0.1311 |
| GSM531302 | IOR-011 | No metastasis | 63 | -1.4139 | 0.6299 |
| GSM531303 | IOR-015 | No metastasis | 60 | -0.8036 | 1.0934 |
| GSM531304 | IOR-016 | No metastasis | 60 | -0.4648 | 0.4192 |
| GSM531305 | IOR-019 | No metastasis | 60 | -1.3477 | 0.7600 |
| GSM531306 | L1016 | Metastasis | 10 | 1.1259 | 0.2834 |
| GSM531307 | L1085 | Metastasis | 39 | 0.0150 | 0.6600 |
| GSM531308 | L1368 | Metastasis | 95 | 0.5861 | -0.0015 |
| GSM531309 | L1369 | Metastasis | 83 | -1.2716 | 1.0236 |
| GSM531310 | L1370 | No metastasis | 246 | 0.1884 | 0.8398 |
| GSM531311 | L1372 | Metastasis | 25 | 0.2600 | 0.0533 |
| GSM531312 | L1376 | Metastasis | 40 | 0.8306 | -0.3643 |
| GSM531313 | L1382 | No metastasis | 143 | 0.2662 | 0.7315 |
| GSM531314 | L1385 | Metastasis | 11 | -0.2763 | -0.3012 |
| GSM531319 | L1386 | No metastasis | 105 | 0.0237 | 0.2146 |
| GSM531320 | L2292 | No metastasis | 78 | -0.8541 | 0.7672 |
| GSM531321 | L2295 | No metastasis | 97 | -0.9985 | 1.5825 |
| GSM531322 | L2296 | Metastasis | 33 | -0.1176 | 0.9066 |
| GSM531323 | L2297 | No metastasis | 77 | -0.4174 | 0.7211 |
| GSM531324 | L2301 | Metastasis | 47 | -0.4656 | 0.7195 |
| GSM531325 | L2302 | No metastasis | 120 | 0.1748 | -0.4188 |
| GSM531326 | L2611 | No metastasis | 91 | -0.1918 | 0.2293 |
| GSM531327 | L2614 | Metastasis | 29 | 1.0125 | -1.3645 |
| GSM531328 | L2616 | Metastasis | 32 | -0.0990 | 0.1457 |
| GSM531329 | L2618 | Metastasis | 31 | 0.6491 | -2.3034 |
| GSM531330 | L2619 | Metastasis | 25 | -0.2207 | 1.1009 |
| GSM531331 | L2620 | No metastasis | 219 | -0.9200 | -0.2311 |
| GSM531332 | L428 | No metastasis | 193 | -0.3088 | 0.4047 |
| GSM531333 | L432 | No metastasis | 184 | -0.1961 | 1.1385 |
| GSM531334 | L436 | No metastasis | 194 | -0.0711 | -0.0455 |
| GSM531335 | L975 | No metastasis | 94 | -0.0324 | 0.8705 |
| GSM531351 | L997 | No metastasis | 87 | 0.4916 | 0.4591 |
| GSM531352 | TJA | No metastasis | 60 | 0.3647 | 0.1951 |

**Supplementary Table S5. Candidate ranks under all weighting scenarios**

| **Gene** | **Balanced** | **Bulk priority** | **Robustness priority** | **Single-cell priority** | **Original integrated rank** |
| --- | --- | --- | --- | --- | --- |
| KIF20A | 1 | 2 | 2 | 1 | 1 |
| ASPM | 2 | 1 | 1 | 6 | 2 |
| KPNA2 | 8 | 7 | 11 | 8 | 3 |
| CCNB2 | 3 | 6 | 6 | 2 | 4 |
| PLK1 | 6 | 9 | 4 | 4 | 5 |
| H2AFX | 12 | 13 | 9 | 19 | 6 |
| TROAP | 5 | 5 | 3 | 11 | 7 |
| TPX2 | 4 | 3 | 7 | 5 | 8 |
| CKS2 | 7 | 4 | 5 | 12 | 9 |
| MKI67 | 9 | 15 | 12 | 3 | 10 |
| CCNB1 | 10 | 10 | 14 | 14 | 11 |
| UBE2T | 20 | 14 | 17 | 31 | 12 |
| CDC25B | 13 | 18 | 8 | 15 | 13 |
| PRR11 | 25 | 27 | 24 | 18 | 14 |
| CDCA3 | 11 | 8 | 10 | 26 | 15 |
| NUSAP1 | 14 | 17 | 18 | 9 | 16 |
| PTTG1 | 28 | 33 | 27 | 17 | 17 |
| HMGB2 | 29 | 30 | 30 | 22 | 18 |
| DEPDC1 | 17 | 19 | 23 | 16 | 19 |
| KIF14 | 15 | 12 | 15 | 25 | 20 |
| NEK2 | 19 | 26 | 20 | 7 | 21 |
| TACC3 | 16 | 11 | 13 | 29 | 22 |
| GTSE1 | 18 | 22 | 16 | 21 | 23 |
| ANLN | 26 | 20 | 21 | 35 | 24 |
| CCNA2 | 27 | 23 | 25 | 34 | 25 |
| CENPE | 22 | 25 | 26 | 13 | 26 |
| HMMR | 21 | 21 | 22 | 20 | 27 |
| CENPA | 30 | 29 | 29 | 27 | 28 |
| BUB3 | 24 | 16 | 19 | 33 | 29 |
| CKAP2 | 31 | 37 | 33 | 23 | 30 |
| HMGB3 | 33 | 35 | 34 | 28 | 31 |
| BIRC5 | 37 | 39 | 35 | 32 | 32 |
| LMNB1 | 34 | 28 | 32 | 40 | 33 |
| CENPF | 23 | 24 | 28 | 10 | 34 |
| PHF19 | 44 | 47 | 44 | 41 | 35 |
| KIF22 | 36 | 31 | 31 | 42 | 36 |
| CKAP5 | 38 | 40 | 38 | 30 | 37 |
| DTYMK | 46 | 46 | 41 | 47 | 38 |
| PRC1 | 35 | 32 | 39 | 36 | 39 |
| UBE2S | 41 | 34 | 36 | 46 | 40 |
| DBF4 | 47 | 44 | 46 | 48 | 41 |
| TOP2A | 32 | 36 | 37 | 24 | 42 |
| RANGAP1 | 45 | 45 | 45 | 43 | 43 |
| RACGAP1 | 43 | 42 | 47 | 45 | 44 |
| CDKN3 | 42 | 43 | 42 | 37 | 45 |
| KIF11 | 39 | 38 | 43 | 38 | 46 |
| KIF2C | 40 | 41 | 40 | 39 | 47 |
| ECT2 | 48 | 49 | 50 | 44 | 48 |
| ANP32E | 50 | 48 | 49 | 50 | 49 |
| LBR | 49 | 50 | 48 | 49 | 50 |

**Supplementary Table S6. Statistical and trajectory audit summary**

| **Audit item** | **Result** |
| --- | --- |
| Pseudobulk design | ~ patient + cell_state; 6 patients; 12 paired libraries; edgeR quasi-likelihood test |
| Pseudobulk reproducibility | 14,292 genes compared; Pearson and Spearman correlations of logFC = 1.000 |
| Module formula | Weighted sum of cohort-standardized expression; all 50 genes found; reconstruction Pearson r = 1.000 |
| Nonmetastatic follow-up | 19/19 patients had at least 60 months of recorded follow-up |
| Slingshot start | M2, selected as the lowest original six-program composite-score cluster |
| Slingshot endpoint | No endpoint supplied; 6 lineages inferred; M2-M8-M6 selected post hoc because it contained M6 |
| Trajectory interpretation | Exploratory transcriptional continuity only; no temporal or causal inference |
